# The semantic richness of abstract concepts

**DOI:** 10.3389/fnhum.2012.00315

**Published:** 2012-11-27

**Authors:** Gabriel Recchia, Michael N. Jones

**Affiliations:** ^1^Department of Cognitive Science, Indiana UniversityBloomington, IN, USA; ^2^Department of Psychological and Brain Sciences, Indiana UniversityBloomington, IN, USA

**Keywords:** concreteness, lexical decision, semantic richness, feature norms, abstract concepts

## Abstract

We contrasted the predictive power of three measures of semantic richness—number of features (NFs), contextual dispersion (CD), and a novel measure of number of semantic neighbors (NSN)—for a large set of concrete and abstract concepts on lexical decision and naming tasks. NSN (but not NF) facilitated processing for abstract concepts, while NF (but not NSN) facilitated processing for the most concrete concepts, consistent with claims that linguistic information is more relevant for abstract concepts in early processing. Additionally, converging evidence from two datasets suggests that when NSN and CD are controlled for, the features that most facilitate processing are those associated with a concept's physical characteristics and real-world contexts. These results suggest that rich linguistic contexts (many semantic neighbors) facilitate early activation of abstract concepts, whereas concrete concepts benefit more from rich physical contexts (many associated objects and locations).

The majority of experimental evidence guiding our knowledge of lexical semantic representation comes from research using concrete words. Concrete words are the dominant stimuli in the literatures on semantic priming, property generation, and single-item recognition (e.g., naming or lexical decision in isolation). In contrast, a larger proportion of the human lexicon may actually be composed of abstract words. For example, of the 500 most frequent words in the TASA corpus (Landauer and Dumais, 1997), 70% are classified as abstract according to the MRC Psycholinguistic Database (Coltheart, 1981). In studies involving large representative samples of English nouns, a substantial proportion (over 40%) are rated as abstract by human raters (Gilhooly and Logie, 1980) and classified as abstract entities in lexical databases such as WordNet (Miller, 1990). Furthermore, abstract words have been implicated as particularly worthy of study due to their status as network hubs (Sigman and Cecchi, 2002), the challenge they pose to grounded theories of cognition (Barsalou, 2008), and their higher relative frequency than concrete words (Audet and Burgess, 1999). In his review of research on grounded cognition, Barsalou (2008) notes “Because the scientific study of concepts has primarily focused so far on concrete concepts, we actually know remarkably little about abstract concepts, even from the perspective of traditional cognitive theories” (p. 634). The disconnect between the type of words we know the most about and the type of words that most inhabit the lexicon means that theoretical development may be over-emphasizing mechanisms and information sources for word representation that do not generalize to the full lexicon.

Abstract concepts are particularly important to study because various theories of semantic representation make different claims about the degree of semantic richness possessed by abstract concepts, particularly with respect to semantic features. Abstract concepts may be semantically impoverished, deriving their meaning primarily from their associations with other words (Paivio, 1986, 2010; Plaut and Shallice, 1993). Alternatively, they may be no less semantically rich, but be more grounded in introspective simulations (Barsalou et al., 2008) or aspects of meaning related to their social/communicative function (Borghi and Cimatti, 2009; Borghi et al., 2011).

If semantic features are important to abstract concept representations, one might expect to find feature-based effects similar to those observed for concrete words. For concrete concepts, being associated with many semantic features facilitates lexical processing (Pexman et al., 2002, 2003; Grondin et al., 2009). These so-called *number of feature* (NF) effects have established the importance of semantic richness in concrete word representation. Investigating whether NF effects are obtained for abstract words—and if so, for what types of features—can yield insight into their representations and the information sources used to learn those representations.

Pexman et al. (2008) found that in addition to NF, a concept's number of semantic neighbors (NSN) and contextual dispersion (CD) accounted for unique response time variance in a lexical decision task. However, their reliance upon the McRae et al. (2005) feature norms to calculate NF restricted their analysis to concrete words. In this paper, we use a novel online game modeled after McRae et al.'s task to gather feature generation data, and present results from data collected from 30 subjects/word for 550 words, including 177 abstract concepts. Extending the methods of Pexman et al. (2008) to this database and to alternative measures of NSN, NF, and CD, we evaluate whether NSN, NF, and CD each account for unique variance in lexical decision times (LDT) for abstract as well as concrete words. We also investigated the specific types of features that contribute to NF effects when NSN and CD are controlled for.

## Are abstract concepts rich in anything?

Several studies have investigated whether the processing and memory advantages often observed for concrete words are due to their allegedly richer featural representations (e.g., Saffran, 1980; Barry, 1984; Plaut and Shallice, 1993; Moss and Tyler, 1995). While there is general agreement that properties of concrete concepts include perceptual and functional features, the literature is less consistent about what exactly qualifies as a property of an abstract concept. When participants are specifically instructed to produce properties that they feel are characteristic of the concept itself, abstract concepts elicit fewer properties than concrete concepts (de Mornay Davies and Funnell, 2000; Tyler et al., 2002). Other studies with a broader definition of what qualifies as a property have found that concrete concepts elicit more properties that explicitly describe the concept (Barsalou and Wiemer-Hastings, 2005; Wiemer-Hastings and Xu, 2005), but have noted that the definition of a property can be extended to include persons, objects, and other elements of situations associated with the concept, as well as internal states and other meaning-bearing utterances. For example, the protocol used by Wiemer-Hastings and Xu classifies the words *good* and *want* in a participant's description of *HOPE* (“something will happen good, you really want something to happen,” p. 736) as words that carry information about internal states (“introspective features”), and many elements of situations were observed in descriptions of abstract concepts in the present study, including mentions of persons (*DANGER* → “a policeman may face this in his job”), objects (*SUCCESS* → “great house”), and events (*MISCHIEF* → “crimes at night”). When information of this sort is not ignored, apparent differences in richness between concrete and abstract concepts disappear or become far less extreme (Wiemer-Hastings and Xu, 2005).

While the present research does tally the number of properties for each concept according to both broad and a narrow criteria, our primary motivation was not to determine whether concrete words possess more properties than abstract ones. Rather, the primary goal was to determine whether the descriptions elicited by abstract words in property generation tasks add to their richness in in a comparable manner to concrete words (i.e., whether “properties” of abstract concepts contribute to NF effects), and if so, what kinds of properties are most facilitative.

On some accounts, the situation-relevant and introspective utterances that participants use to describe abstract concepts in feature generation tasks are conceived of as properties in a strong sense, playing a central role in abstract concept representations (Barsalou and Wiemer-Hastings, 2005; Barsalou et al., 2008). If this is the case, one might expect that the quantity of introspective and situation properties that an abstract word elicits would predict its ease of processing, just as the number of perceptual properties does for concrete words (Grondin et al., 2009).

However, such utterances may not describe core components of the concept's representation at all. One possibility is that the words that participants use to describe abstract concepts are analogous to *associates*, i.e., participants' responses in free-association tasks. Studies of concrete concepts that have directly compared the influence of NF and *number of associates* (NoA) have shown the latter to have a relatively weak or undetectable impact (Yap et al., 2011; Rabovsky et al., 2012), and the same may be true for abstract concepts. A second possibility is that the words that participants use to describe abstract concepts may facilitate processing to the degree that they occur in similar linguistic contexts. It has been argued that language-based information plays a more important role in abstract (vs. concrete) concept representations (e.g., Sabsevitz et al., 2005; Borghi et al., 2011). NSN measures the number of words (NW) that occur in similar lexical contexts (Pexman et al., 2008), as approximated by counting the NW that occur within a particular radius of a high-dimensional semantic space. Such language-based measures of richness have been shown to predict LDT among concrete concepts (Buchanan et al., 2001; Pexman et al., 2008; Yap et al., 2011). According to theories that emphasize the importance of linguistic information for abstract concepts, such measures of the richness of a word's linguistic contexts should be even more predictive of processing differences among abstract stimuli.

Of course, this need not be framed as a dichotomy. For example, Kiefer and Pulvermüller (2011) argue that multimodal information from perception and action constitutes the core content of abstract concept representations, but also note that abstract concepts may be more dependent upon on word-based associations than concrete concepts. Furthermore, a measure of semantic richness is not wholly language-based merely because it is derived from a text corpus; word pairs that are highly related according to corpus-based measures such as LSA frequently refer to objects that occur together in the world (Baroni and Lenci, 2008; Louwerse, 2008). Even so, the fact that measures based on corpora and feature norms account for unique variance in lexical and semantic decision tasks suggests that corpus-based measures contain some information about associations between words as they are used in language that feature norms do not, and vice versa (see Riordan and Jones, 2011). Contrasting the predictive power of multiple measures of richness can thus inform our understanding of the relative importance of different types of information to concrete and abstract conceptual representations.

## Measures of semantic richness

The three variables considered by Pexman et al. (2008)—NF, CD, and NSN—are not the only ones that have been investigated as measures of semantic richness. Yap et al. (2011) extended this work in several ways. First, they included additional variables that had been proposed in the literature as indicators of semantic richness: NoA (Duñabeitia et al., 2008) in the Nelson et al. (1998) free-association norms, and *lexical ambiguity*, which they operationalized as a word's log-transformed number of senses in WordNet (Miller, 1990). Second, they used alternative CD and neighborhood measures that had been calculated on larger corpora and accounted for more variance than previous operationalizations of CD and NSN.

Finally, they included additional lexical control variables known to account for substantial variance in lexical decision and naming times (NTs). Using these measures, they found that neighborhood density, CD, and NF all accounted for unique variance above and beyond that accounted for by lexical-level variables in the lexical decision task, whereas number of senses and lexical ambiguity did not. Yap et al. also found that CD and NF, but not NSN, predicted unique variance in speeded pronunciation times, although the effects were less robust. This is consistent with previous findings of facilitation for words with many features (Ashcraft, 1978; Pexman et al., 2003), contexts (Adelman et al., 2006; Jones et al., 2012), and semantic neighbors (Buchanan et al., 2001; Shaoul and Westbury, 2010).

## Collecting semantic richness norms for concrete and abstract words

Our norming study builds on Yap et al. (2011) by replicating their pattern of effects for lexical decision and speeded pronunciation on a new set of stimuli containing words that vary widely in concreteness. The absence of publicly available feature norms for English abstract concepts required us to collect a large set of property generation norms. In a property generation study, participants describe the properties of a concept verbally or in writing. For example, presented with the concept *dog*, participants might produce descriptions such as *has four legs*, *is furry*, etc. This method has a long history of use by researchers wishing to gain insight into the representations of concrete concepts and categories (e.g., Rosch and Mervis, 1975; Hampton, 1979; McRae et al., 2005; Vinson and Vigliocco, 2008), and less frequently, events and abstract concepts (e.g., Barsalou and Wiemer-Hastings, 2005; Wiemer-Hastings and Xu, 2005; Vinson and Vigliocco, 2008). Property norms should not be interpreted as a verbatim readout of semantic representations (Medin, 1989), but rather as a reflection of systematic regularities in the ways that participants describe concepts. They can provide insight into a concept's underlying semantic representation, although not all aspects of meaning are equally well represented. Some aspects of a concept's representation are not easily verbalized, while others may be particularly salient due to their distinctiveness. This poses philosophical challenges to traditional views that interpret features as fundamental components of semantic representations. However, it is less problematic for positions that treat features as offering a window into aspects of semantic meaning (McRae et al., 2005) or as *ad-hoc* descriptions of perceptual simulations (Barsalou and Wiemer-Hastings, 2005). McRae et al. (2005) note that although the absence of biological features, internal features, etc., is “occasionally interpreted as a weakness of such norms, it may actually be a strength, because it appears that these general features play only a small role in object identification, language comprehension, and language production precisely because they are not salient and are true of large numbers of concepts” (p. 549). Overall, the impressive ability of measures based on feature norms to account for variability across a wide range of lexical processing tasks (e.g., Yap et al., 2012) attests to their utility in capturing important aspects of meaning.

Following the basic design of McRae et al. (2005), our participants completed a property generation task in the form of an online game within our *Semantic Pictionary* platform (Kievit-Kylar and Jones, 2011). Online “games with a purpose” (von Ahn, 2006) are becoming more commonplace in cognitive science to crowdsource information about the properties of common objects from Internet users (e.g., Singh et al., 2002; Speer et al., 2010). This method permitted participants to describe abstract words without constraining them to produce properties in the form of predicates such as *has wings*, *is fast*, etc. There is a high correlation between *ease of predication* (participants' ratings of how easy it is to put words into simple factual statements) and concreteness (Jones, 1985; de Mornay Davies and Funnell, 2000), leading some to surmise that abstract words have far fewer properties than concrete words (e.g., Plaut and Shallice, 1993). If a property is defined as a predicate, this is a foregone conclusion.

However, predicates are not the basic units of semantic analysis, but are rather only one way of expressing underlying semantic relationships. *Wing*, *passenger*, and *pilot* are all concepts that possess a meaningful semantic relationship to *airplane*, and language affords us an easy way to express these relationships as predicates (airplanes *have* wings, airplanes *carry* passengers, airplanes *need* pilots). However, *courthouse*, *crime*, and *justice* are all concepts that possess a meaningful semantic relationship to *law*, and may play a role in its semantic representation, even if it is difficult to produce a three- or four-word sentence that encapsulates the nature of this relationship. We risk missing important insights about the nature of abstract concept representation if we exclude such concepts from analysis simply because participants do not express them as predicates. On the other hand, if we interpret all frequent responses as “features,” we risk being too inclusive. We do not pretend to have a solution to this dilemma, and believe there may be value in both broad and constrained notions of what constitutes a property. For this reason, we restricted our definition of NF to the number of {concept → word} pairs that matched a subset of predefined semantic relations identified in the literature as being of likely importance to concrete and abstract concept representations. However, we also created an additional variable, NW, which is simply a count of all words produced by at least six of the 30 subjects who generated descriptions for that word. Details on how each of these measures was calculated appear in the Methods section.

### Methods

#### Participants

Seven hundred and sixty six participants (57% female) participated via the Indiana University Psychology Department subject pool for partial course credit. An additional 208 participants recruited via Amazon Mechanical Turk who completed the study for a payment of $1 per session. All participants resided in the United States and reported English as their first language.

#### Materials

After surveying the literature on feature generation studies and abstract word representation, 593 English nouns were selected to be normed. Items used in the feature generation studies of Barsalou and Wiemer-Hastings (2005), McRae et al. (2005), Wiemer-Hastings and Xu (2005), and Vinson and Vigliocco (2008) were selected to facilitate comparison between the data to be collected and that collected by other researchers, to build upon previous findings that used existing datasets, and because these items were originally selected to represent a broad range of stimuli used in the semantic memory literature. Additional stimuli were selected from the MRC Psycholinguistic Database (Coltheart, 1981) in order to ensure our stimuli included words with a high level of diversity in frequency, length, and concreteness. All words were classified for concreteness/abstractness on the basis of their rated MRC concreteness (see Analysis 2). See Recchia and Jones (2012) for the complete set of stimuli.

#### Procedure

***Property generation***. Participants were asked to participate in an online game in which they would be required to describe various words, and were informed that a future participant would be responsible for guessing the words from their descriptions. Participants were asked to provide 10 short descriptive properties for each of 20 words that would help their partner guess the target word. Participants were instructed to describe the concept, not the word itself; i.e., clues about the letter that a word starts with or words that it rhymed with were not permitted. Participants were asked to fill in all 10 blanks, but the online application did not require all 10 blanks to be filled in order to move on to the next word. Word type was alternated for each participant (i.e., each concrete word followed an abstract word and vice versa), and word type that began the task was balanced across participants. While McRae et al. (2005) provided explicit instructions about the sorts of properties they wanted subjects to produce (“physical properties, such as internal and external parts, and how it looks, sounds, smells, feels, or tastes; functional properties, such as what it is used for; where, when and by whom it is used; things that the concept is related to, such as the category that it belongs in; and other facts, such as how it behaves, or where it comes from,” McRae et al., p. 556), our instructions left this considerably more open-ended, asking participants to “provide 10 short descriptive properties for each word that will help your partner guess your noun, without specifically telling your partner which word you have,” with further instructions emphasizing that participants were responsible for describing the concept, not surface features of the word itself (e.g., “rhymes with”). At the completion of the study, 93% of the original 593 stimuli had been described by at least 30 participants, the same number of participants per word recruited by McRae et al. (2005); words for which this was not the case were excluded from analysis. The resulting set of 550 words included 281 items from the McRae et al. norms.

***Measures of semantic richness***. Four measures of semantic richness were obtained for each of the 550 cue words: NW, NF, NSN, and CD. Each concept's NW was determined by counting the number of unique words (types) produced by at least six^1^ of the 30 subjects who generated descriptions for that concept. The set of words produced by at least six participants in response to a given concept were reformatted as a list of {concept → word} pairs. Some pairs exhibited a clear semantic relationship that could be expressed as a predicate (e.g., {key → metal}: *is made of*), while others exhibited semantic relationships that were not necessarily expressed as predicates but were captured by Wu and Barsalou's (2009) taxonomy of semantic codes for generated properties (e.g., {danger → emergency}: *event*). Yet others matched categories not covered in the Wu and Barsalou taxonomy, but which have been hypothesized to be of particular importance to abstract concept representations, such as communicative acts and social institutions/artifacts (Barsalou and Wiemer-Hastings, 2005; Wiemer-Hastings and Xu, 2005; Borghi and Cimatti, 2009).

Although Wu and Barsalou (2009) reported high levels of rater agreement for concrete words, our initial attempts at using their taxonomy for our set of abstract words proved relatively unreliable, as did our initial attempts to use taxonomies developed for coding free-response protocols (Barsalou and Wiemer-Hastings, 2005; Wiemer-Hastings and Xu, 2005). After multiple rounds of classification of a subset of properties by two raters, we ultimately settled on the partial taxonomy detailed in Recchia and Jones (2012). It is not intended to represent a complete set of feature types, as it omits several property types hypothesized to be highly relevant to concrete word representations (e.g., functions; agentive actions; category coordinates). The primary reason for this was that we wished to include only those feature types for which high levels of rater agreement could be achieved. Are *bowl* and *spoon* best conceived of as *category coordinates* (e.g., eating utensils) or *associated entities*? Systematic disagreements of this nature between raters were generally solved either by collapsing multiple categories into one or omitting a category entirely. However, when fine-grained distinctions could be preserved while retaining high reliability, we generally did so. Agreement between two raters on a 500-feature subset of the data was quite good (Cohen's κ = 0.78), and so the remainder of the data was coded by a single rater. Each {concept, word} pair that was produced by at least six participants and which matched one of these codes was considered a feature and was included in the NF measure.

A common trade-off in the development of a coding scheme is between reliability and comprehensiveness; our criteria clearly lean toward reliability. Our exclusion of some feature categories means that some valid features will have escaped our NF measure, but the feature categories that are coded for are consistent between raters. Thus, our NW and NF measures represent broad and narrow ends of the spectrum of definitions of what constitutes a “feature.” As with any measure of NF, it is important to keep in mind that exactly how features are defined is critically important to the interpretation of NF effects (or the absence thereof).

Finally, NSN was calculated for each concept. Pexman et al. (2008) used global semantic neighborhood values calculated by Durda et al.'s (2006) *WordMine2* application. According to this measure, a word's neighborhood consisted of all words occurring within a specific radius of the high-dimensional space defined by HAL (Lund and Burgess, 1996), a co-occurrence-based model of lexical semantics. Yap et al. (2011) replaced this with an alternative measure of corpus-based neighborhood density that reflected the mean cosine between a word and its 5000 closest neighbors in a HAL-like semantic space (Shaoul and Westbury, 2010). In both studies, while high NSN facilitated performance in lexical decision, NSN had null effects on semantic decision tasks. Each study noted that this was perhaps due to the fact that neighborhood measures conflate close and distant neighbors, which have opposite effects on processing in some circumstances (Mirman and Magnuson, 2008). Yap et al. (2011) suggested that this could be partially addressed by parametrically manipulating the number of neighbors considered. In addition, the size of the window used for assessing whether the nearby appearance of two words counts as a “co-occurrence” should be treated as a parameter that must be optimized (Bullinaria and Levy, 2007).

Rather than compute a definition of NSN theoretically tied to a vector space model such as HAL, we calculated NSN using pointwise mutual information (PMI), a measure of association frequently used in computational linguistics to contrast the actual probability of observing two items together (e.g., in the same window of text) with the probability of having observed them together if they had been independently distributed (Manning and Schütze, 1999). PMI is calculated as

(1) $$\log_{2}\frac{p(xy)}{P(x)P(y)}$$

where *P*(*x*) represents the probability of observing word *x* if a random window of text is selected from the corpus, *P*(*y*) the probability of observing word *y*, and *P*(*xy*) the probability of observing *x* and *y* together. PMI has been shown to be a good predictor of human semantic similarity and synonymy judgments (Recchia and Jones, 2009), and allows for a straightforward manner of calculating a measure of NSN not tied to any particular semantic space model: one can simply count the NW having a PMI exceeding some threshold *t*, using a window size of *w*. Exploratory manipulation of these parameters indicated that using the TASA corpus (Zeno et al., 1995), a window size of 8 and a threshold of 7 maximized correlations between this measure of NSN and LDT, and that the same parameters maximized correlations to NTs as well. These were therefore the parameters used for the calculation of NSN in the analyses reported here.

Finally, following Yap et al. (2011), a measure of CD was obtained from the English Lexicon Project (Balota et al., 2007), and the number of senses attributed to each word by lexicographers was obtained from WordNet (Miller, 1990). What is referred to in the following analyses as CD refers specifically to log SUBTL-CD, the logarithm of the number of transcribed film and television programs in the SUBTLEX corpus (Brysbaert and New, 2009) in which a word appears.

## Exploring the semantic richness norms

In this section, we conduct three basic analyses to explore the effects of semantic richness in our norms on lexical decision and naming data extracted from Balota et al. (2007). First, we attempt to replicate the overall findings of Yap et al. (2011) using our larger norms that have greater variability in concreteness. Second, we repeat the analysis separately for the abstract and most concrete sets of words in our norms to evaluate whether facilitative effects of semantic richness are consistent across the two words types. Finally, we expand our NF variable into counts of particular *types* of features to determine which features are most responsible for explaining the variance in response latency, and whether these responsible feature types differ between concrete and abstract words.

### Analysis 1: replicating Yap et al. (2011)

To attempt to replicate the effects obtained by Yap et al. (2011) with our dataset and our measures of NF and NSN, we conducted a hierarchical regression analysis to assess the impact of measures of semantic richness on lexical decision and NTs. We used a near-identical set of control variables to Yap et al., but omitted Coltheart's *N* and its analog for phonological neighbors, as these are measures designed to account for the same underlying construct as the improved Levenshtein distance measures (orthographic/phonological density). Thus, the control variables entered into the regression were log-frequency (SUBTLEX corpus), number of morphemes, number of syllables, orthographic Levenshtein distance 20 (Yarkoni et al., 2008), and phonological Levenshtein distance 20. To control for phonetic biases in voice key response time measurements, a set of dichotomous onset variables taking on values of 0 or 1 for each stimulus were used to code for the absence/presence of 13 phonetic features (Balota et al., 2007; Yap et al., 2011, 2012); these were entered as additional predictors in the regression analysis of NT latencies only.

The measures of semantic richness entered were NW, NF, NSN, and CD, described in the preceding section^2^. Z-scores of LDT and NTs were obtained from the English Lexicon Project (Balota et al., 2007). Control variables were also obtained from this dataset; these were entered in the first step of the regression, while measures of semantic richness were entered in the second step. Descriptive statistics for each of these variables are presented in Table 1. All 550 stimulus items were included in the regression.

**Table 1:** **Descriptive statistics for stimulus characteristics (predictors and dependent variables)**.

	**All 550 stimulus items**	**Set of 147 abstract items**	**Set of 147 most concrete items**
	***M* (SD)**	***M* (SD)**	***M* (SD)**
Rated concreteness (MRC norms)	5.07 (1.37)	3.21 (0.49)	6.24 (0.11)
**CONTROL VARIABLES**			
Log frequency (Brysbaert and New, 2009)	2.75 (0.76)	3.15 (0.82)	2.86 (0.63)
Number of morphemes	1.27 (0.53)	1.48 (0.68)	1.09 (0.31)
Number of syllables	1.78 (0.84)	2.03 (0.98)	1.60 (0.76)
Number of letters (length)	5.85 (1.97)	6.35 (2.09)	5.29 (1.80)
OLD20 (Yarkoni et al., 2008)	2.12 (0.84)	2.18 (0.68)	1.97 (0.79)
PLD20 (Yarkoni et al., 2008)	1.96 (0.94)	2.05 (0.84)	1.76 (0.88)
**SEMANTIC RICHNESS VARIABLES**			
Number of semantic neighbors	150.61 (97.77)	199.59 (91.00)	167.08 (96.40)
Number of features (Analysis 1)	11.08 (3.83)	8.56 (3.78)	13.00 (3.24)
Number of features (McRae et al., 2005)	14.13 (3.64)	–	15.40 (3.59)
Number of words (Analysis 1)	32.10 (7.47)	27.36 (7.26)	35.61 (6.33)
Log ctx. dispersion (Brysbaert and New, 2009)	2.51 (0.68)	2.91 (0.67)	2.59 (0.57)
Log number of senses (Miller, 1990)	1.32 (0.82)	1.57 (0.79)	1.26 (0.75)
**DEPENDENT MEASURES**			
Lexical decision task RT (Balota et al., 2007)	653.16 (84.53)	644.35 (78.55)	630.71 (74.49)
Lexical decision task RT, standardized	−0.48 (0.28)	−0.52 (0.27)	−0.56 (0.26)
Pronunciation task RT (Balota et al., 2007)	635.37 (61.17)	634.23 (58.83)	624.97 (49.73)
Pronunciation task RT, standardized	−0.42 (0.26)	−0.42 (0.26)	−0.47 (0.22)

Note: OLD20, Orthographic Levenshtein Distance 20, a measure of orthographic neighborhood density; PLD20, Phonological Levenshtein Distance 20, a measure of phonological neighborhood density. The number of features measure in the McRae et al. (2005) dataset limited to those concepts that were used as stimuli in our dataset and which were also members of the McRae norms (first column, N = 281; third column, N = 93). Average rated concreteness for the entire stimulus set was computed over only the 446 words for which MRC concreteness ratings were available.

#### Results

As anticipated, NF, NSN, and CD were each found to be independent predictors of variance in LDT (*p* < 0.001) even after variance from control variables had been accounted for. Table 2 reports correlations between each pair of variables, while Table 3 reports betas and *p*-values from the regression analysis. Consistent with Yap et al. (2011), CD remained a significant predictor for NTs while NSN dropped out^3^, although we did not find our measure of NF to predict NTs.

**Table 2:** **Intercorrelations among predictor and dependent variables in the regression analyses for all stimuli**.

	**NSN**	**NF_RJ_**	**NF_M^†^_**	**NW**	**CD**	**Freq**	**Nm**	**Ns**	**Len**	**OLD**	**PLD**	**Sens**	**LDT_raw_**	**LDT_Z_**	**NT_raw_**	**NT_Z_**
NSN	–	−0.199^**^	0.189^**^	0.055	0.729^**^	0.686^**^	−0.035	−0.191^**^	−0.201^**^	−0.311^**^	−0.307^**^	0.496^**^	−0.519^**^	−0.551^**^	−0.366^**^	−0.373^**^
NF_RJ_		–	0.238^**^	0.475^**^	−0.169^**^	−0.133^**^	−0.163^**^	−0.031	−0.057	0.068	0.051	−0.307^**^	0.009	0.011	−0.004	0.013
NF_M^†^_			–	0.098	0.219^**^	0.232^**^	0.057	−0.093	−0.089	−0.081	−0.089	0.037	−0.235^**^	−0.243^**^	−0.102	−0.119^*^
NW				–	0.095^*^	0.093^*^	−0.150^**^	−0.139^**^	−0.131^**^	−0.075	−0.083	0.005	−0.113^**^	−0.129^**^	−0.130^**^	−0.124^**^
CD					–	0.985^**^	−0.157^**^	−0.314^**^	−0.354^**^	−0.435^**^	−0.422^**^	0.582^**^	−0.634^**^	−0.685^**^	−0.507^**^	−0.514^**^
Freq						–	−0.195^**^	−0.333^**^	−0.379^**^	−0.442^**^	−0.430^**^	0.562^**^	−0.623^**^	−0.673^**^	−0.496^**^	−0.504^**^
Nm							–	0.541^**^	0.621^**^	0.465^**^	0.502^**^	−0.230^**^	0.291^**^	0.323^**^	0.305^**^	0.291^**^
Ns								–	0.820^**^	0.756^**^	0.804^**^	−0.386^**^	0.506^**^	0.527^**^	0.466^**^	0.451^**^
Len									–	0.871^**^	0.852^**^	−0.375^**^	0.559^**^	0.598^**^	0.551^**^	0.558^**^
OLD										–	0.916^**^	−0.457^**^	0.570^**^	0.607^**^	0.510^**^	0.519^**^
PLD											–	−0.442^**^	0.547^**^	0.587^**^	0.499^**^	0.511^**^
Sens												–	–	–	–	–
LDT_raw_													–	0.949^**^	0.647^**^	0.642^**^
LDT_Z_														–	0.684^**^	0.682^**^
NT_raw_															–	0.948^**^
NT_Z_																–

Note: NSN, number of semantic neighbors; NF_RJ_, number of features, Analysis 1; NF_M_, number of features, McRae et al. (2005); NW, number of words; CD, log contextual dispersion; WF, log word frequency; Nm, number of morphemes; Ns, number of syllables; Len, number of letters; OLD, Orthographic Levenshtein distance 20; PLD, Phonological Levenshtein distance 20; Sens, log number of senses; LDT_raw_, lexical decision time; LDT_Z_, lexical decision time (z-scored); NT_raw_, naming time; NT_Z_, naming time (z-scored).

† NF_M_ has a large number of missing values due to the fact that only 281 words in the McRae et al. (2005) norms also appear in the present dataset.

** p < 0.01;

* p < 0.05.

**Table 3:** **Standardized regression coefficients predicting lexical decision and naming latencies, using number-of-features measure derived from data collected in Analysis 1**.

**Variables**	**Betas**	
	**LDT**	**NT**
**STEP 1: ONSETS**		
Adjusted *R*^2^	0.00	0.17^***^
**STEP 2: CONTROL VARIABLES**		
Log frequency	−0.505	−0.352^***^
Number of morphemes	−0.036	−0.065^†^
Number of syllables	0.071	0.059
Number of letters (length)	0.286^***^	0.407^***^
OLD20	0.105	−0.075
PLD20	−0.009	0.060
Adjusted *R*^2^	0.59	0.55
Change in *R*^2^	0.59^***^	0.38^***^
**STEP 3: SEMANTIC RICHNESS VARIABLES**		
Number of features	−0.114^***^	−0.036
Number of words	0.027	−0.021
Number of semantic neighbors	−0.135^***^	−0.027
Log contextual dispersion	−0.848^***^	−1.038^***^
Log number of senses	−0.051	0.034
Adjusted *R*^2^	0.63	0.58
Change in *R*^2^	0.04^***^	0.03^***^

Note: LDT, lexical decision time (z-scored); NT, naming time (z-scored); OLD20, Orthographic Levenshtein Distance 20, a measure of orthographic neighborhood density; PLD20, Phonological Levenshtein Distance 20, a measure of phonological neighborhood density. Only semantic richness variables are shown in Step 2 for ease of exposition.

† p < 0.10;

*** p < 0.001.

#### Discussion

Despite differences in our stimuli and in our measures for NF and NSN, we found a pattern of effects consistent with those reported elsewhere in the literature—particularly for lexical decision—giving us confidence that our measures tap into semantic richness constructs similar to those investigated by other researchers. However, this in itself tells us nothing about whether different types of richness contribute differentially to the processing of abstract vs. concrete concepts, as the majority of our dataset consisted of concrete concepts. In Analysis 2, we examine whether the same pattern of effects holds for the most abstract and most concrete words in our dataset.

### Analysis 2: semantic richness predictions for concrete vs. abstract words

Consistent with prior research, Analysis 1 found unique contributions of NF, NSN, and CD in lexical decision. Do we observe differential effects for concrete and abstract words? If NSN and NF pattern differently for words of different levels of concreteness, this would lend support to theories that predict differences in the involvement of language in abstract and concrete representations.

Two regressions were conducted using the same methods described in Analysis 1, but were restricted to the abstract stimuli and an equally sized subset of the most concrete stimuli rather than the entire set (Recchia and Jones, 2012). MRC concreteness ratings were available for 446 of the 550 stimuli used in the feature generation task. Of these, 147 met the criteria used by Wiemer-Hastings and Xu (2005) to define abstractness (MRC rating lower than 4.5; see Wiemer-Hastings and Xu, 2005, Appendix A). This set of 147 abstract concepts was contrasted with a set of the 147 most concrete concepts (stimuli with the highest MRC ratings). Table 1 provides descriptive statistics for the dataset as a whole and for the abstract/concrete subsets. Hierarchical regressions were computed with the same sets of control and semantic richness variables as in the previous analysis.

#### Results

Table 4 reports betas and *p*-values for the variables predicting lexical decision and naming latencies. For the set of 147 abstract words, NSN and CD were found to be significant predictors of LDT (*p* < 0.05), but NF was not (*p* = 0.15). In contrast, for the set of the 147 most concrete items, NF (*p* < 0.01) and CD (*p* < 0.001) were found to be significant predictors of LDT, but NSN was not (*p* = 0.14). When LDT regressions were repeated with one or neither Levenshtein distance predictor, there were no changes in which semantic richness variables remained significant and non-significant predictors. With Levenshtein variables omitted, variance inflation factors (VIFs) were acceptably low for both abstract concepts (4.0 and 1.8 for control and semantic variables, respectively) and concrete concepts (3.3 and 3.0). We considered the possibility that NSN was not a significant predictor for the most concrete words merely because the variance accounted for by NF and NSN overlapped in such a way that NSN would have been a significant predictor for the most concrete words had NF not been part of the analysis. However, NSN was still not a significant predictor for the set of the most concrete words even when NF was omitted from the regression (*p* = 0.2). Similarly, NF was not a significant predictor for the set of abstract words even when NSN was omitted from the regression (*p* = 0.4).

**Table 4:** **Standardized regression coefficients predicting lexical decision and naming latencies**.

**Variables**	**Betas**			
	**Set of 147 abstract items**		**Set of 147 most concrete items**	
	**LDT**	**NT**	**LDT**	**NT**
**STEP 1: ONSETS**				
Adjusted *R*^2^	0.00	0.14^**^	0.00	0.25^***^
**STEP 2: CONTROL VARIABLES**				
Log frequency	−0.413^***^	0.331^***^	−0.514^***^	−0.291^***^
Number of morphemes	0.094	−0.013	−0.080	−0.136^*^
Number of syllables	−0.004	−0.195	−0.098	0.025
Number of letters (length)	0.444^**^	0.575^***^	0.223^†^	0.234
OLD20	0.053	−0.231^†^	0.066	−0.186
PLD20	−0.119	0.309^*^	0.238	0.313^†^
Adjusted *R*^2^	0.54	0.54	0.62	0.53
Change in *R*^2^	0.54^***^	0.40^***^	0.62^***^	0.28^***^
**STEP 3: SEMANTIC RICHNESS VARIABLES**				
Number of features	−0.089	0.072	−0.168^**^	−0.115^†^
Number of words	0.024	0.008	0.083	0.006
Number of semantic neighbors	−0.147^*^	−0.072	−0.121	−0.060
Log contextual dispersion	−0.890^*^	−1.094^*^	−0.944^***^	−1.069^**^
Log number of senses	−0.043	0.045	−0.024	0.045
Adjusted *R*^2^	0.59	0.58	0.67	0.57
Change in *R*^2^	0.04^***^	0.04^***^	0.05^***^	0.04^***^

Note: LDT, lexical decision time (z-scored); NT, naming time (z-scored); OLD20, Orthographic Levenshtein Distance 20, a measure of orthographic neighborhood density; PLD20, Phonological Levenshtein Distance 20, a measure of phonological neighborhood density. Only semantic richness variables are shown in Step 2 for ease of exposition.

† p < 0.10;

* p < 0.05;

** p < 0.01;

*** p < 0.001.

For NTs, CD was the only significant semantic richness predictor for abstract and concrete concepts. NF was a marginally significant predictor for concrete concepts (*p* = 0.07), but not for abstract concepts (*p* = 0.28).

#### Discussion

As previously described, different theories of concept representation make different predictions with respect to the role of language and semantic features for abstract concepts. Internal experiences (felt experiences of judgments, cognitive operations, emotional valence, etc.) have been hypothesized to play a special role in grounding abstract concepts, as have complex situations involving multiple actors, particularly social actors (Barsalou and Wiemer-Hastings, 2005; Wiemer-Hastings and Xu, 2005; Borghi and Cimatti, 2009). Indeed, the set of abstract concepts under investigation was rich in these categories of features. Abstract concepts were relatively high in several categories of features hypothesized by these researchers to be of particular importance, with an average NF per concept of 0.66 for communicative acts (vs. 0.07 for concrete concepts), 0.61 for evaluations (vs. 0.34 for concrete concepts), 1.35 for social artifacts/actions (vs. 0.26 for concrete concepts), and 2.6 for cognitive states/operations/affects—a higher mean than in any single feature category for the most concrete concepts, although concrete concepts elicited more features overall.

The fact that abstract concepts were so frequently described in terms of internal and social experiences hints that these may indeed be important aspects of abstract concept representation. However, the present analyses suggest that being rich in these kinds of features likely does not facilitate early processing of abstract concepts in the same way that being feature-rich facilitates early processing of highly concrete concepts. Of course, it is certainly possible that annotating participants' descriptions for other kinds of features would yield different results. It is also possible that 147 abstract concepts was simply too few to detect a NF effect. However, the fact that NF was a significant predictor of the 147 most concrete concepts' LDT (*p* < 0.01), and approached significance in the NT regression^4^ (*p* = 0.07), implies a stronger role for features in concrete concept representations.

Another way in which our results differed between abstract and concrete concepts was in the degree of facilitation provided by NSN. In cross-task comparisons, semantic neighborhood density has been shown to facilitate concrete concept processing in lexical decision, but not in other tasks such as semantic decision or word naming (Yap et al., 2012). Analysis 1 replicated this pattern of results: Using a large dataset consisting of primarily concrete concepts, NSN was found to be a significant predictor of lexical decision but not NTs. In Analysis 2, however, no effect of NSN was found on a smaller dataset consisting of only the most concrete concepts. This may have been due to the loss of statistical power resulting from the smaller subset of stimuli (147 items). However, the fact that NSN *was* a significant predictor for an equally small set of abstract concepts suggests an important dissociation.

Given that NSN represents the richness of the linguistic contexts in which words denoting particular concepts appear, a greater role for NSN in abstract than concrete concept representations is consistent with hypotheses that abstract representations are more heavily grounded in language than are concrete representations, whether researchers attribute this to differences in the process by which abstract concept meanings are acquired (Della Rosa et al., 2010; Borghi et al., 2011) or to abstract concept representations' purported dearth of multimodal perceptual information (Plaut and Shallice, 1993; Paivio, 2010). This finding is not necessarily inconsistent with theories that ground both concrete and abstract concepts in non-linguistic content (e.g., Barsalou et al., 2008), as long as these theories can be extended to explain why measures of language-based richness are more predictive of LDT for abstract words than for concrete ones. Theories in which abstract concepts are represented primarily as conceptual metaphors (Lakoff, 2009) would also require additional scaffolding to accommodate this result.

So far, these analyses do not tell us *which* features are facilitating the processing of concrete words, nor if there are subsets of features that may be differentially facilitating abstract word processing. For example, situation properties, social institutions/artifacts (Barsalou and Wiemer-Hastings, 2005), and internal experiences (Barsalou et al., 2008) have been argued to be central to abstract word representations, as have aspects of meaning related to social/communicative function (Borghi and Cimatti, 2009; Borghi et al., 2011). Does the number of such specific properties generated in a feature generation task predict LDT for abstract words? This issue is investigated in the final analysis.

### Analysis 3: what types of features facilitate processing of concrete vs. abstract words?

Although our composite NF variable did not predict lexical decision or NTs for the set of abstract concepts considered in Analysis 2, perhaps the number of particular kinds of features would have proved to be reliable predictors if they had been considered as separate variables. In addition, it seems likely that not all types of features are equally important for the NF effects observed for concrete concepts. Features representing differing knowledge types have been shown to follow different timecourses of activation (Amsel, 2011), some of which are protracted enough that it is unlikely that they would have an influence on lexical decision. The purpose of Analysis 3 was to investigate what types of features account for the lion's share of the variance predicted by NF, and whether this differed between abstract and concrete concepts.

### Analysis 3a: fine-grained semantic categories

NF was decomposed into 19 separate variables, each of which represented the NFs of a particular type (e.g., number of locations, number of visual properties, etc.) detailed in Recchia and Jones (2012), and the regressions from Analyses 1 and 2 were repeated. Descriptive statistics on the NFs in each category are displayed in Table 5. Three separate regressions were again conducted on all stimuli, on abstract stimuli only, and on most-concrete stimuli only, using the same methods as in Analyses 1 and 2, but with NF broken into 19 separate predictors representing the NFs of different semantic types. VIFs for the set of semantic richness variables remained low after these transformations, with max VIFs of 2.9, 2.0, and 3.8 for the set of all stimuli, abstract stimuli, and most-concrete stimuli, respectively, and VIFs of each of the 19 new predictors less than 1.7. Furthermore, because only LDT showed a reliable effect of NF in Analyses 1 and 2, NTs were not included as a dependent measure.

**Table 5:** **Descriptive statistics for type counts of different feature categories**.

	**All 550 stimulus items**	**Set of 147 abstract items**	**Set of 147 most concrete items**
	***M* (SD)**	***M* (SD)**	***M* (SD)**
Num. communicative acts (com)	0.28 (1.00)	0.66 (1.31)	0.07 (0.48)
Num. materials (has_material)	0.49 (0.92)	0.01 (0.12)	0.77 (1.24)
Num. components (has_part)	1.17 (1.60)	0.04 (0.23)	1.67 (1.66)
Num. larger continuous wholes (is_material_of)	0.21 (0.76)	0.00 (0.00)	0.56 (1.23)
Num. larger discrete wholes (is_part_of)	0.08 (0.45)	0.01 (0.08)	0.16 (0.67)
Num. visual properties (vis)	0.50 (0.88)	0.09 (0.33)	0.73 (1.06)
Num. non-visual perceptual properties (perc)	1.35 (1.55)	0.05 (0.21)	2.07 (1.54)
Num. cognitive states/operations/affects (cog)	1.00 (1.98)	2.61 (3.05)	0.27 (0.51)
Num. contingencies (conting)	0.07 (0.29)	0.16 (0.42)	0.05 (0.24)
Num. evaluations (eval)	0.42 (0.84)	0.61 (1.16)	0.34 (0.66)
Num. negations (neg)	0.31 (0.52)	0.48 (0.63)	0.21 (0.44)
Num. social artifacts/actions (soc)	0.58 (1.35)	1.35 (2.01)	0.26 (0.59)
Num. events (ev)	0.23 (0.57)	0.19 (0.46)	0.27 (0.71)
Num. locations (loc)	0.85 (1.15)	0.29 (0.80)	1.20 (1.17)
Num. manners (man)	0.05 (0.23)	0.05 (0.21)	0.07 (0.26)
Num. participants (par)	0.64 (0.96)	0.78 (1.05)	0.62 (0.95)
Num. associated entities (ae)	1.48 (1.51)	0.65 (1.09)	1.71 (1.45)
Num. times (time)	0.31 (0.81)	0.39 (1.00)	0.21 (0.43)
Num. super/subordinates (tax)	1.06 (1.20)	0.14 (0.40)	1.75 (1.25)
**SUPERCATEGORIES**			
Num. entity properties	3.80 (3.30)	0.20 (0.51)	5.97 (2.74)
Num. introspective properties	1.79 (2.42)	3.86 (3.40)	0.86 (0.94)
Num. taxonomic properties	1.06 (1.20)	0.14 (0.40)	1.75 (1.25)
Num. concrete situation properties	2.33 (1.89)	0.95 (1.40)	2.91 (1.65)
Num. other situation properties	1.23 (1.47)	1.41 (1.59)	1.18 (1.30)

Although we coded our own data according to our own feature taxonomy, we also wished to take advantage of the fact that the McRae et al. (2005) feature norms have been similarly annotated with a fine-grained set of semantic relations. Specifically, the WB_Label field labels each of McRae et al.'s 7259 {concept, feature} pairs with one of 27 categories nearly identical to those described in Wu and Barsalou (2009, Appendix A). Because these norms constitute a separate dataset to which a separate set of feature codes has been applied by other raters, we hoped that including a comparable analysis that utilized this dataset might offer complementary insights as to which feature types most strongly drive NF effects. We therefore also repeated the regression conducted in Analysis 1 on the subset of 281 concepts that occurred in the McRae et al. (2005) norms as well as our own stimuli, replacing NF with 27 separate variables, each of which represented the NFs in the McRae et al. norms of a particular WB_Label type (i.e., internal components, locations, and the 25 other feature types appearing in their WB_Label column). These were not highly intercorrelated, with semantic richness variables exhibiting a max VIF of 4.1, and the 27 new variables' VIFs being less than 2.0 in all cases.

#### Results

For the regression conducted on the set of 147 abstract words, none of the 19 new NF variables accounted for unique variance in LDT. Similarly, for the set of 147 most concrete words, none of the 19 NF variables individually accounted for unique variance in LDT. However, for the full set (analog to Analysis 1), the number of the following kinds of features accounted for unique variance: *locations* (locations in which the concept is found; *p* < 0.001), *associated entities* (objects that tend to co-occur in real-world situations with the concept; *p* < 0.05), and *larger continuous wholes* (objects that are made out of a material described by the concept; *p* < 0.05). No other feature classes were a significant predictor of unique variance in our data. Betas and significance levels for this analysis are reported in Recchia and Jones (2012).

For the regression using the feature counts, semantic classes, and data from McRae et al. (2005), *locations* (*p* < 0.05) and *associated entities* (*p* < 0.001) were the only two variables that explained significant unique variance. No other feature classes accounted for unique variance in this dataset. The full set of betas and significance levels for this analysis are listed in Recchia and Jones (2012).

#### Discussion

Our replications of Analysis 2 with specific feature types proved inconclusive: When restricting the regressions to 147-concept subsets and dividing NF into 19 variables, none of the variables accounted for significant levels of variance in LDTs, although this could be due to data sparsity. However, on the two replications of Analysis 1 using our data and the data from McRae et al. (2005), there was converging evidence that NF effects for concrete words are primarily driven by *locations* and *associated entities*. Although both location and associated entity features were relatively common, their predictive power does not seem to derive solely from their overall high frequency. For example, several classes of properties (non-visual perceptual properties, components, subordinates/superordinates, cognitive states/operations) were more frequent than locations.

The finding that *locations* and *associated entities* were predictive in both datasets is especially striking, considering the substantial differences in the process according to which features were coded in each. As previously described, the NF measure in the McRae et al. norms is a count of the number of distinct predicates used to describe each concept, whereas our measure of NF was obtained for each concept by counting the number of distinct {concept → word} pairs that matched a set of predefined semantic relations. Although an investigation of three publicly available sets of properties (Howell et al., 2005; McRae et al., 2005; Vinson and Vigliocco, 2008) and two sets of associates (Kiss et al., 1973; Nelson et al., 1998) indicated that our concept vectors were more highly correlated with those of the McRae norms than were those of the other datasets (Recchia et al., 2011), the correlation between the *number of associated entity* variables in the two regressions conducted in Analysis 3a is weak (*r* = 0.24). This is likely due to differences in the way the category of *associated entity* was defined in our coding categories vs. the Wu and Barsalou (2009) categories used in the McRae norms (see General Discussion). Thus, one might reasonably argue that the *number of associated entity* variables in these regressions tap different constructs, although the fact that both are estimates of the number of distinct objects that occur together with the concept in real-world situations is suggestive. However, the *location* categories in the two coding systems have very similar definitions, and the correlation between the *number of location* variables is substantially higher (*r* = 0.44). The fact that *number of locations* accounts for unique variance across multiple datasets and coding schemas suggests that being associated with many physical contexts facilitates lexical decision latencies for concrete concepts.

Surprisingly, variables such as *number of visual properties* did not predict LDT, even though shared visual form/surface properties have predicted LDT in at least one previous study (Grondin et al., 2009). Our failure to detect an effect may have been due in part to the high fractionation in feature types, resulting in low statistical power. Our final analysis again replicates Analysis 1, but groups features into the four supercategories proposed by Wu and Barsalou (2009): entity properties (physical or systemic properties of the entity itself, such as visual properties), situation properties (properties of situations in which the entity occurs), introspective properties (properties of mental states associated with the concept), and taxonomic properties (hypernyms, hyponyms, etc.). The two feature types that predicted unique variance in Analysis 3 (locations and associated entities) are unique among situation properties in that they pick out *concrete objects*—i.e., they answer the question, “what things co-occur with this concept?” As such, they are distinguished from other situation properties in the following analysis.

### Analysis 3b: coarse-grained semantic categories

All regressions performed in Analysis 3a were repeated, with two differences: First, the 27 NF variables corresponding to feature subtypes in the McRae norms were grouped into five supercategories representing the number of *entity properties*, *introspective properties*, *taxonomic properties*, *concrete situation properties* (associated entities and locations), and *other situation properties*. See Wu and Barsalou (2009) for the taxonomy of which subtypes belong in which supercategories. Second, the feature subtypes in our own norms were similarly reclassified according to their reference number in Recchia and Jones (2012) as *entity properties* (2, 3, 4, 5, 6, 7), *introspective properties* (8, 9, 10, 11), *taxonomic properties* (19), *concrete situation properties* (14, 17), and *other situation properties* (13, 15, 16, 18). Means and standard deviations for the NFs in each supercategory are reported in Table 5.

#### Results

In our own norms, the supercategory variables that predicted lexical decision latency were *number of entity properties* (*p* < 0.05), *number of concrete situation properties* (*p* < 0.01), and *number of other situation properties* (*p* < 0.05). Predictive supercategory variables for the McRae subset were *number of entity properties* (*p* < 0.05) and *number of concrete situation properties* (*p* < 0.01). The correlation between *number of entity properties* variables calculated using the McRae data/codes vs. our own data/codes was 0.41. Tables 6 and 7 report betas and significance levels for these analyses.

**Table 6:** **Standardized regression coefficients predicting lexical decision latencies, using feature counts and codes from data collected in Analysis 1**.

**Variables**	**Betas**
**STEP 1: CONTROL VARIABLES**	
Log frequency	−0.505^***^
Number of morphemes	−0.036
Number of syllables	0.071
Number of letters (length)	0.286^***^
OLD20	0.105
PLD20	−0.009
Adjusted *R*^2^	0.59
**STEP 2: SEMANTIC RICHNESS VARIABLES**	
Number of words	0.056^†^
Number of semantic neighbors	−0.130^***^
Log contextual dispersion	−0.940^***^
Log number of senses	−0.045
Num. entity properties	−0.074^*^
Num. introspective properties	−0.037
Num. taxonomic properties	−0.051
Num. concrete situation properties	−0.098^**^
Num. other situation properties	−0.060^*^
Adjusted *R*^2^	0.63
Change in *R*^2^	0.04^***^

Note: OLD20, Orthographic Levenshtein Distance 20, a measure of orthographic neighborhood density; PLD20, Phonological Levenshtein Distance 20, a measure of phonological neighborhood density. Only semantic richness variables are shown in Step 2 for ease of exposition.

† p < 0.10;

* p < 0.05;

** p < 0.01;

*** p < 0.001.

**Table 7:** **Standardized regression coefficients predicting lexical decision latencies, for all stimuli used in Analyses 1–2 that occur in the McRae et al. (2005) norms, using feature counts and codes from the McRae et al. (2005) dataset**.

**Variables**	**Betas**
**STEP 1: CONTROL VARIABLES**	
Log frequency	−0.538^***^
Number of morphemes	−0.081^†^
Number of syllables	0.083
Number of letters (length)	0.299^**^
OLD20	−0.006
PLD20	0.051
Adjusted *R*^2^	0.62
**STEP 2: SEMANTIC RICHNESS VARIABLES**	
Number of words	−0.007
Number of semantic neighbors	−0.021
Log contextual dispersion	−0.450^†^
Log number of senses	−0.075
Num. entity properties	−0.083^*^
Num. introspective properties	−0.002
Num. taxonomic properties	−0.005
Num. concrete situation properties	−0.101^**^
Num. other situation properties	0.017
Adjusted *R*^2^	0.64
Change in *R*^2^	0.02^***^

Note: OLD20, Orthographic Levenshtein Distance 20, a measure of orthographic neighborhood density; PLD20, Phonological Levenshtein Distance 20, a measure of phonological neighborhood density. Only semantic richness variables are shown in Step 2 for ease of exposition.

† p < 0.10;

* p < 0.05;

** p < 0.01;

*** p < 0.001.

As before, no supercategory variables predicted lexical decision latencies for the set of 147 abstract stimuli. In contrast, *number of entity properties* predicted lexical decision latencies for the set of the 147 most concrete nouns, *b* = −0.167, *p* = 0.002.

#### Discussion

As expected from the results of Analysis 3a, the number of *concrete situation properties* (associated entities and locations) was a strong predictor of lexical decision latency in both datasets. In addition, the number of *entity properties* facilitated lexical decision, a fact obscured by the many subcategories in the previous analysis. *Entity properties* are properties of the concept itself, both physical (visual, auditory, etc.) and systemic (e.g., the concept's components, or entities of which it is a component); they are extremely infrequent for abstract concepts–see the General Discussion and Recchia and Jones (2012), categories 2–7 for examples. One might argue that *number of entity properties* reached significance merely because it was the most frequent property type. However, *number of introspective properties* did not predict unique variance for the 147 abstract stimuli, despite the fact that introspective properties were more frequent for this group than *number of entity properties* were for the group of the most concrete stimuli (Table 5). In contrast, *number of entity properties* did predict unique variance for the most concrete stimuli (*p* = 0.002). Overall, *entity* and *concrete situation* properties appear to drive NF effects, consistent with the finding of Analysis 2 that NF predicted LDT for concrete but not abstract words.

## General discussion

We replicated the general findings from Pexman et al. (2008) and Yap et al. (2011) that NSN, NF, and CD all account for unique variance in LDT. However, repeating this analysis for only the abstract words and for an equally sized subset of the most concrete words, NSN (but not NF) facilitated processing for abstract words, while NF (but not NSN) facilitated processing for the most concrete words. As Yap et al., (2011, 2012) noted, results for NTs were generally attenuated and did not show reliable effects of NF or NSN. However, length, log frequency, and CD generally predicted significant levels of variance for naming as well as LDT. Due to the correlation that Levenshtein distance measures (OLD20, PLD20) shared with each other and with log frequency, log frequency was not always a reliable predictor of LDT when these variables were included in the regression, but it was a consistent predictor when one or both of these variables were omitted.

With respect to the types of features that drive NF effects in lexical decision, we did not find evidence to suggest that having a high number of introspective, situation properties, social, or communication-related properties facilitated the processing of abstract or concrete words. This may have simply been due to data sparsity, as no fine-grained feature type predicted LDT in the analyses that were restricted to the 147 abstract or the 147 most concrete stimuli in the dataset. However, when analyzing our entire set of stimuli, we found a similar pattern of effects across two sets of feature norms—ours and those of McRae et al. (2005)—showing in each case, that the number of *entity properties* and *concrete situation properties* (*locations* and *associated entities*) that were attributed to a concept predicted its lexical decision latency. *Entity properties* generally refer to physical characteristics of objects, and are far more frequent for concrete words (*M* = 3.80, SD = 3.30) than for abstract words (*M* = 0.20, SD = 0.51). Generally speaking, entity properties were only attributed to abstract words that were capable of being visualized or audiated despite being rated as abstract (e.g., hell → **brimstone**; crash → **loud**), or which had structured components (story → **plot**). *Locations* are references to places in which the concept might be located; examples from our data include helicopter → **air**, raisin → **box**, pigeon → **city**, cancer → **lung**, etc. These, too, were quite frequent for concrete nouns (Table 5). Examples for abstract concepts were rare, but did occur, e.g., ache → **tooth**, thought → **head**, heaven → **clouds**, plea → **court**, etc.

Associated entities are similar to locations in that they pick out entities that co-occur with the concept in real-world situations. However, the category is significantly broader: an associated entity need not *be* the location in which the concept is located, but it may occur *in* the same location as the concept in the real-world. Examples include *squirrel* → ***acorns***, *beach* → ***castles***, etc. Note that in these particular examples, there is no similarity relation: squirrels are not similar to acorns, and beaches are not similar to castles. However, our raters found that in many cases, it was extremely difficult to disentangle these notions, as many items that are similar to each other also tend to occur in similar contexts (*comb* → ***brush***, *broccoli* → ***cauliflower***, *wall* → ***ceiling***, *spoon* → ***fork***, etc.). Therefore, we did not attempt to distinguish the two types of relation, but collapsed them both under a single category: as noted in Recchia and Jones (2012), we defined code 17 as “An object similar to the entity, or tending to co-occur with the concept in real-world situations.” The definition of *associated entity* in the Wu and Barsalou coding scheme that was used to code the McRae norms is considerably narrower: “an entity in a situation that contains the focal concept” (Wu and Barsalou, 2009, p. 187). Despite these differing definitions, the number of associated entities per concept predicted LDT when analyzing both our data/codes and the data/codes of McRae et al.

Given that NF facilitated processing for the set of the 147 most concrete concepts, but not for the set of 147 abstract concepts, and that the full dataset consisted primarily of concrete concepts, it seems likely that being rich in locations, associated objects, and salient physical characteristics (entity properties) facilitates lexical decision for concrete concepts. It is inconclusive whether this is the case for abstract concepts. One intriguing similarity between locations and associated entities is that each picks out concrete entities (places or objects) that co-occur with the concept in day-to-day situations. This suggests that the features that facilitate concrete concept processing include those that pick out a concept's real-world contexts (cf. Hare et al., 2009). The consistency in the pattern of effects observed suggests that NF's ability to predict unique LDT variance owes at least in part to the fact that it captures the number of places and objects associated with a concept in the real-world.

In contrast, rich *linguistic* contexts (many semantic neighbors) appear to facilitate early activation of abstract concepts, as demonstrated in Analysis 2. This may be due to the fact that we acquire and use abstract words primarily in social situations in which language is highly salient (Borghi and Cimatti, 2009), or that we have no choice but to ground abstract words in language definitions because they have no perceptual referents (Paivio, 1986), or because language use encodes information about both abstract and concrete words (Louwerse, 2008) and the information so encoded is simply more relevant to abstract concept semantics. Given that NSN predicted LDTs for the entire dataset—composed primarily of concrete words—semantic density certainly seems to have a role to play for concrete concepts. However, Analysis 2 demonstrates that the *relative* influence of NF appears to be greater than that of NSN for the most concrete words, whereas the reverse appears to be true for abstract words. Furthermore, this dissociation does not seem to be an artifact of overlapping variance: For the most concrete words, NSN remained insignificant even when NF was removed from the regression, whereas for the most abstract words, NF remained insignificant even when NSN was removed from the regression. This was as expected, given the low correlation between NF and NSN.

While these results do not rule out the possibility that abstract words are simply grounded in different sorts of features than are concrete words, it appears that features of the kind we have investigated in this study do not provide the same sort of processing advantage for abstract as for concrete words. This is perhaps not surprising, given the shallowness of processing that is required for lexical decision—simulation of emotions, internal states, communication-related words, etc., may indeed prove facilitative in tasks requiring deeper processing. Future directions will investigate the influence of NF and NSN on tasks requiring greater depth of processing, such as semantic decision, and test alternative coding schemes for the classification of abstract features.

### Alternative tasks

Although semantic richness effects can be detected across a variety of tasks, task-specific differences can reveal important insights into the structure of semantic memory. In studies using concrete stimuli, NF effects have been observed for standard lexical decision, go/no-go lexical decision, progressive demasking, and semantic classification, while semantic neighborhood density only accounts for unique variance in standard and go/no-go lexical decision (Pexman et al., 2008; Yap et al., 2011, 2012). This difference has been attributed to feedback from orthography, as well as to differences in task demands. Because lexical decision requires only a familiarity judgment, the more neighbors the better: every additional neighbor serves as evidence that the target is in fact a word, and the combined activation of many such neighbors speeds the decision (as long as such neighbors are sufficiently distant, cf. Mirman and Magnuson, 2008). If linguistic associates are a core part of the representations of abstract concepts in a way that they are not for concrete ones, NSN may pattern differently for abstract words on deep processing tasks such as semantic classification.

Alternatively, if features represent *ad-hoc* verbal descriptions of the content of simulations and simulations for abstract concepts are activated relatively slowly due to their greater complexity (Barsalou and Wiemer-Hastings, 2005), this could provide an alternate explanation of why NF facilitates lexical decision for concrete but not abstract stimuli. If this is the case, then NF might facilitate semantic classification of abstract concepts, even though it had no measurable effect on abstract concept LDTs. Further study with a greater variety of semantic tasks could shed light on these intriguing questions.

### Alternative codes

Any classification scheme that attempts to shoehorn a rich conceptual space into a set of discrete and non-overlapping categories faces significant limitations, and ours is no exception. For example, in the property generation task conducted by Barsalou and Wiemer-Hastings (2005), utterances tagged with code EVC (“any act of communication,” p. 159) were far more frequent in participants' descriptions of abstract concepts, and there is some evidence to suggest that abstract concepts may be primarily grounded in acts of communication (Borghi and Cimatti, 2009; Della Rosa et al., 2010; Borghi et al., 2011). Therefore, we wished to include a category (Code 1) encompassing communicative acts (e.g., *explain, demand, call, shout*) and entities with a communicative purpose (e.g., *instructions*, *messages*, *conversation, recommendation, argument*), as this seemed likely to be a type of feature that might participate in abstract concept representations. As many abstract concepts are themselves communicative terms, this category often overlapped with code 19: taxonomic superordinates/subordinates. Due to the taxonomic ambiguity of these terms (is an *inquiry* a kind of *request*?) and the relatively low theoretical relevance of taxonomic relationships to abstract concept representations, such conflicts were resolved by defining code 19 as “hypernyms and hyponyms not otherwise coded.” These were seemingly sensible decisions that resulted in high interrater reliability due to ease of coding: All words that described communicative acts or entities with a communicative purpose were tagged with code 1. However, it also meant that the *communication* category became populated with a mishmash of synonyms (yell → **holler**), hypernyms (rule → **decree**), functions (phone → **communicate**), terms that occur when participants describe situations relevant to the concept or its opposite (truth → **lie**), etc. This example alone should make it clear that many possible alternative coding schemes are possible. Although we found no influence of NF on lexical decision for abstract words, alternative methods of subdividing the feature space may reveal feature categories for which a higher NFs does facilitate abstract LDTs.

### Alternative approaches

While it is possible that we did not detect NF effects for abstract concepts due to the wrong task or the wrong codes, it is also possible that counting features is simply not a useful method for uncovering the structure of abstract concepts. Indeed, our finding that rich linguistic contexts facilitate LDT moreso for abstract words than for highly concrete words is consistent with theoretical claims that language plays a central role in abstract concept representations (Paivio, 1986; Crutch and Warrington, 2005; Borghi et al., 2011), as well as with neuroimaging meta-analyses showing greater activation in language areas during abstract concept processing (Binder et al., 2009; Wang et al., 2010). What might such language-based representations look like? One promising answer comes from corpus-based models of semantic memory such as LSA (Landauer and Dumais, 1997), which construct semantic representations on the basis of distributional statistics. Several computational modelers have demonstrated that superior performance can be achieved on various tasks by extending distributional models with sensorimotor information for concrete concepts (Howell et al., 2005; Jones and Recchia, 2010; Steyvers, 2010; Johns and Jones, 2012); abstract concepts are indirectly grounded in such models by virtue of their linguistic relationships with (grounded) concrete concepts. Alternatively, the corpus-based model of Vigliocco et al. (2009) directly grounds abstract concepts in a combination of linguistic and affective information.

Other approaches include the work of Schmid (2000), who conducted an intensive corpus-based study that elucidates connections between the syntactic and semantic properties of a wide range of abstract nouns and presents an in-depth taxonomy of abstract concept types. Yet others have investigated abstract concepts using such diverse lenses as metaphor (Lakoff, 2009), force dynamics (Talmy, 1988), and many others (see Pecher et al., 2011, for a review). Feature generation should be considered merely one of many possible tools for investigating the nature of abstract concept representations.

## Conclusion

Questions about the role of context in abstract concept representation go back at least as far as Schwanenflugel and Shoben (1983). Ultimately, the greatest benefit of feature norms for a large set of abstract and concrete concepts may be a better understanding of the precise role that context plays in scaffolding word meaning. The present studies suggest that, at least in lexical decision, NSN facilitates activation of abstract concepts, while NFs facilitates activation of concrete concepts. Analysis of two datasets of feature generation data provided converging evidence that the number of *entity properties* and *concrete situation properties* (i.e., *locations* and *associated entities*) primarily drove our NF effects. A broad interpretation of these results consistent with some theories of concept representation is that while rich language contexts facilitate abstract concept recognition, rich physical characteristics, and contexts facilitate concrete concept recognition. Similar investigations using different tasks are likely to add further nuance to our understanding of different forms of semantic richness and the conceptual representations they support.

### Conflict of interest statement

The authors declare that the research was conducted in the absence of any commercial or financial relationships that could be construed as a potential conflict of interest.

## Footnotes

^1^This was the same threshold used by Vinson and Vigliocco (Andrews et al., 2009), and is nearly identical to the 5-subject threshold used by McRae et al. (2005).

^2^Like Yap et al. (2011, 2012), we did not include NoA as a predictor in our primary regression analyses due to the fact that NoA counts were available for only some of our stimuli, as this would have reduced the power of our analyses substantially. When the regressions reported in this paper were repeated with NoA included as a predictor, NoA was not significant in any analysis.

^3^Also consistent with Yap et al. was a failure to find any contribution of orthographic and phonological Levenshtein distance, most likely because of the overlapping variance accounted for by these two predictors. Indeed, the high correlation between orthographic and phonological Levenshtein distance gave the set of control variables a maximum VIF (variance inflation factor) of 8.3, indicating concerning levels of multicollinearity. However, omitting either (or both) of these predictors did not change which semantic richness variables predicted significant levels of variance; the only change in the pattern for control variables was that log-frequency became a significant predictor of lexical decision times (*p* < 0.01), a finding more consistent with the known influence of frequency on lexical decision times.

^4^For the regression using naming times of concrete concepts as the dependent variable, two unexpected results were the extraordinarily high amount of variance accounted for by the phonetic onset variables (adjusted *r*^2^ = 0.25) and the lack of significance for word length (Table 4). Closer inspection of the data suggested that this was likely the result of a chance correlation between word length and onset characteristics. Of the 13 dichotomous phonetic onset variables, the one having the strongest point-biserial correlation with word length was the *approximant* variable (*r*_pd_ = −0.16, *p* = 0.06); this variable also accounted for the most variance (compared with other onset variables) among naming times in the regression (*b* = −0.46, *p* = 0.005). In other words, our concrete stimuli that began with approximants happened to be particularly short words, which may have caused phonetic onsets to appear to be stronger predictors of variance than they truly were.
